# Research on the Application Value of Intelligent Heating Box in Newborn Nursing

**DOI:** 10.1155/2021/7081995

**Published:** 2021-12-06

**Authors:** Yongzhi Lu, Guangrong Bo, Yuanyuan Hu

**Affiliations:** Department of Neonatology, The Hospital of Suixi County, Suixi, Anhui 235100, China

## Abstract

The aim of this study was to explore the application effect of intelligent incubator in neonatal care. We selected the period from January 1, 2018, to December 31, 2020, where there were 100 full-term and premature babies born in a hospital and transferred to the neonatal intensive care unit (NICU) within 1 hour after birth. Before the improved heat preservation, 100 full-term infants in the control group and 100 full-term infants in the intervention group of the intelligent warming box were formed into a full-term infant group for a comparative study. Statistics and comparison of the two groups of term infants and premature infants admitted to the hospital were carried out to assess body temperature and the changes in the incidence of each system. The research found that on comparison of admission body temperature between the control group and the intervention group, with the intervention group in the intelligent heating box, the incidence of hypothermia was significantly lower than that of the control group (95% vs. 37% of full-term infants; 98% vs. 49% of premature babies; there is a statistical significance (*P* < 0.05)). The intelligent heating box can reduce the fluctuation of the newborn's body temperature, keep the internal environment of newborns stable, and provide suitable conditions for the rapid growth of newborns, suitable for clinical promotion and application.

## 1. Introduction

Clinically, a baby is called a newborn when it is less than 28 days old, under external stimuli, due to its low physical fitness and immunity; therefore, it is extremely prone to various diseases [1]. In the process of nursing newborns, taking reasonable and effective measures to take care is particularly critical; it can not only improve the various symptoms of newborns but can also effectively reduce its various complications [2]. In recent years, as people's health awareness and health awareness continue to increase, the assessment and care of the newborn's health at birth have been paid more and more attention to [3]. At present, the society is paying more and more attention to neonatal epidemiological research, the cesarean section rate in a city from 2018 to 2020 is as high as 44.35%, the rate of premature babies is as high as 11.82%, the perinatal mortality rate is as high as 5.30%, and the premature infant rate and perinatal mortality rate are increasing year by year [4]. So, both parents and medical staff put forward higher requirements for the clinical care of newborns. Therefore, professional and systematic nursing guidance is adopted to improve the quality of newborn care, and it is very necessary to promote the normal growth and development of newborns [5, 6].

The 2016 American Academy of Pediatrics Guidelines for Neonatal Resuscitation states: for newborns who have not suffocated after birth, body temperature after birth should be kept between 36.5∼37.5°C. It is recommended that the medical staff should also record the body temperature of the newborn during the resuscitation process and as one of the indicators to evaluate the quality of recovery. It is recommended to use in addition to the radiation bed for the insulation of newborns, cotton hats should also be used, polyethylene plastic film should be used to wrap the newborn's body, as it can heat the mattress and warm and humidify oxygen, at the same time, a series of comprehensive heat preservation measures such as ensuring the indoor temperature are needed to carry out the heat preservation work of newborns after birth, especially to avoid hypothermia in premature babies, and while keeping warm, it is also important to avoid excessive body temperature rise (>38°C) and reduce the potential adverse effects caused by high temperature [7]. There are rare reports of full-term children and reports on the adverse effects of hypothermia on term infants and premature infants. Thompson, T. S., and others introduced the development of neonatal intensive care unit (NICU) patients, the human-centered design strategy of solutions for families and staff, to prepare from the open bay (OB) NICU to a separate room (SFR) NICU. Through a series of user group meetings, the Nichols family, administrators, providers, an interdisciplinary team of nurses, and other nursing team members (CTMS), in collaboration with design professionals, created and carried out their vision for the new NICU. This process spans the design of the project, construction and transition plans, enabling stakeholders of the Medical University of South Carolina, South Carolina (USA), to seek solutions that address patient and home center care into fabrics with its new facilities and redesign the nursing experience. From this work, new opportunities for families and employees have emerged. Continuous end-user participation leads to targeted preparations for neonatal care [8].

In summary, after treating newborns born in the past 5 years in a hospital with traditional and simple heat preservation methods and intelligent warm box heat preservation methods, we observe and summarize the entry temperature of full-term infants and premature infants, vital signs and response to hypoglycemia, oxygen demand, respiratory failure, neonatal wet lungs, acidosis, RDS, feeding intolerance, delayed meconium discharge, gastrointestinal bleeding, neonatal hypoxic-ischemic encephalopathy, and changes in the incidence and mortality of multiple systems such as intracranial hemorrhage. The purpose of this topic is to summarize an effective heat preservation measure and sum up the experience for the newborn heat preservation process in our hospital in the future. At the same time, it summarizes the data on the incidence of full-term infants and premature infants under comprehensive and effective insulation measures and lays the foundation for future scientific research.

## 2. Research Methods

### 2.1. Research Objects

The selected research objects of this topic are selected from January 1, 2018, to December 31, 2020; live-born full-term infants and premature infants born in a hospital and transferred to the neonatal intensive care unit (NICU) within 1 hour after birth were the subjects of the study.

#### 2.1.1. Inclusion Criteria

(1) All live-born newborns who were transferred to the neonatal ward within 1 hour after delivery in the delivery room and operating room. (2) The gestational age is limited to 29–41 weeks.

#### 2.1.2. Exclusion Criteria

(1) The mother has other serious diseases such as fever and infection (such as gestational diabetes and uncontrolled thyroid dysfunction). (2) The newborn has various congenital diseases (congenital heart disease, septal disease, etc.) and congenital malformations after birth, such as lack of organs and limbs and severe skin damage (such as spina bifida and umbilical bulge). (3) Although transferred to the neonatology department, the family members transferred to the hospital on their own or gave up treatment due to their illness or family members. All newborns in the abovementioned standard items are banned from being selected for this topic.

### 2.2. Method

#### 2.2.1. Grouping Method

From January 1, 2018, to June 30, 2019, a total of 200 newborns (including 100 full-term infants and 100 premature infants) were randomly selected as the control group among all newborns born before the use of intelligent incubators. From July 1, 2016, to December 31, 2018, a total of 200 newborns (including 100 full-term infants and 100 premature infants) were randomly selected as the intervention group among all newborns born after using the intelligent incubator.

#### 2.2.2. Control Group Insulation Measures

All newborn infants in the control group were given routine heat preservation measures, including cleaning the respiratory tract on the preheated radiation table (34°C) in the delivery room or operating room, after the basic operations such as breaking the umbilical cord, drying the body, weighing, and wrapping it in a quilt, and the child was transferred to the neonatal ward by medical staff.

#### 2.2.3. Insulation Measures of the Intervention Group

On this basis, for the intervention group, comprehensive insulation measures were adopted, a number of prevention methods were added for hypothermia, and the specific process is as follows: ①first, we prepare for resuscitation, including prenatal consultation; understanding the condition of the newborn, a team of pediatric medical staff and obstetric medical staff will form a resuscitation team and conduct equipment inspections at the same time; and the the temperature of the delivery room and operating room is controlled at 25–26°C; ②the transport incubator is preheated to 34°C in advance, and all the related supplies that will be in contact with the child, such as wet wipes, baby diapers, finger pulse oximetry, the blood pressure cuff, and the auscultation head of the stethoscope, are put into the preheated incubator to preheat and reserve; ③the radiant table is preheated in advance, and the temperature of the radiation table is controlled to 34°C; the newborn's cotton cap is put on the radiant bed to preheat, at the same time, for newborns to wear after birth; ④the double-layer cotton sheet is preheated on the radiant table at the same time (the first layer is used to dry the body, and the second layer is used to wrap the newborn's body); ⑤for premature infants, especially very-low-birth-weight infants and ultra-low-birth-weight infants, it is made sure to prepare the plastic film needed to wrap the newborn; ⑥after the newborn is delivered and the umbilical cord is broken, the newborn is immediately wrapped and dried with the first layer of warm blanket, the newborn is wrapped with the second layer of warm blanket; ⑦if it is a very-low-birth-weight infant, the whole body will not dry; instead, it quickly wraps the newborn's head with a warm plastic film, and the trunk and lower limbs prevent heat loss; ⑧when weighing, a preheated blanket is put on the scale and weighed quickly; the preheated woolen cap is put on the newborn; ⑨body temperature within 10 minutes after birth is measured and recorded in the delivery room; and ⑩the newborn is transferred to the NICU with a transfer incubator; the incubator is made to enter immediately after entering the NICU; and body temperature within 10 minutes of admission is measured.

### 2.3. Statistical Methods

SPSS23.0 statistical software was used for data analysis. Normally distributed measurement data are represented by $\bar{X}\pm s$, and counting data are expressed in proportion (%). The measurement data of the two groups were compared by the *t*-test, and the *X*^2^ test was used to compare count data. The comparison of the grade data uses an anecdotal test. All data test results are statistically significant with *P* < 0.05.

## 3. Result Analysis

### 3.1. Comparison of Basic Data between the Two Groups of Research Objects

In terms of full-term infants, the intervention group with an intelligent heating box and the control group with simple heat preservation measures contained 100 full-term infants. Between the two groups of full-term newborns, at gestational age 38.5 ± 1.1 weeks vs. 38.4 ± 1.0 weeks, birth weight 3341 ± 403 g vs. 3316 ± 293 g, and age 25 ± 3 min vs. 24 ± 3 min, there was no significant difference in gender (male/female) and cesarean section rate (62% vs. 60%) (*P* > 0.05).

In terms of premature babies, the intervention group with an intelligent heating box and the control group with simple heat preservation measures contained 100 premature babies. Between the two groups of premature infants, at gestational age 32.5 ± 1.6 weeks vs. 32.2 ± 1.9 weeks, birth weight 1680 ± 354 g vs. 1616 ± 310 g, and age 24 ± 3 min vs. 23 ± 4 min, there was no significant difference in gender (male/female) and cesarean section rate (58% vs. 52%) (*P* > 0.05).

### 3.2. The Incidence of Admission to Hospital and the Distribution of Admission Body Temperature of Term Infants and Premature Infants

**Figure 1:**
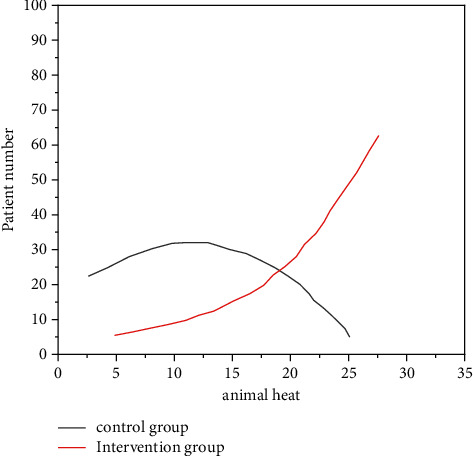
The distribution of body temperature of full-term infants admitted to the hospital.

**Figure 2:**
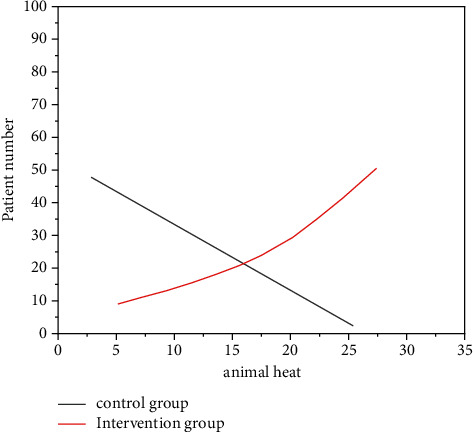
The distribution of body temperature of premature infants admitted to the hospital.

Analyzing and comparing (Table 1) the intervention group after the intelligent incubator, the incidence of hypothermia was significantly lower than that of the control group (term infants 95% vs. 37%; 98% vs. 49% of preterm infants), statistically significant (*P* < 0.05). The distribution of the two groups of newborns in different temperature intervals is shown in Figure 1 and Figure 2.

### 3.3. Comparison of Neonatal Morbidity between the Two Groups

#### 3.3.1. Comparison of the Incidence of Term Infants between the Two Groups (See Table 2)

In this study, it was found that, in full-term children, comparing the intervention group and the control group, after comprehensive and effective heat preservation, in the demand for oxygen, the incidence of neonatal wet lung showed a significant decrease, and the comparison between the two groups showed statistical differences (*P* < 0.05). In aspiration pneumonia and respiratory failure, the incidence of RDS has a decreasing trend, but it has not yet constituted a statistical difference (*P* > 0.05). After admission, there was no significant difference in the incidence of bradycardia (<100 beats/min) between the two groups (*P* > 0.05); in terms of admission blood test indicators, hypoglycemia and metabolic acidosis are significantly reduced, and there are statistical differences (*P* < 0.05). Abnormal coagulation indicators, hyperbilirubinemia, electrolyte disturbances, increased HCT, thrombocytopenia, etc., although in a trend of decreasing incidence, can be observed; however, this group of studies has not yet constituted a statistical difference (*P* > 0.05). At the same time, this group's research found that, with the significant decrease in the incidence of hypothermia, the number of Apgar <7 points in 1 minute or 5 minutes is also reduced accordingly and showed a statistical difference (*P* < 0.05), and it is suggested that the incidence of hypothermia is positively correlated with the incidence of asphyxia [9, 10]. In terms of neurological diseases, although the number of cases of neonatal H, E, and intracranial hemorrhage decreased slightly in this group of data, however, there was no statistical difference in this group of studies (*P* > 0.05). In the digestive system, the incidence of feeding intolerance and gastrointestinal bleeding was slightly reduced to a certain extent, but neither constituted a statistical difference (*P* > 0.05).

#### 3.3.2. Comparison of the Incidence of Premature Infants between the Two Groups (See Table 3)

In premature infants, the incidence rate between the intervention group and the control group is more significant than that of the term infant group; in many systems, the incidence rate has been significantly reduced, and more system diseases showed statistical differences [11]. For example, there are significant differences in bradycardia (heart rate <100 beats/min), Apgar <7 points in 1 minute or 5 minutes after admission, and there are statistically significant differences (*P* < 0.05); at the same time in the respiratory system, the incidence of oxygen demand, neonatal wet lung, and apnea in the intervention group was significantly reduced, and there were statistical differences (*P* < 0.05); although aspiration pneumonia, RDS, and respiratory failure showed a downward trend in this group of studies, there was no statistical difference (*P* > 0.05). Digestive system: in many aspects such as feeding intolerance, delayed meconium discharge, and gastrointestinal bleeding, the intervention group was also significantly different from the control group, and the difference was statistically significant (*P* < 0.05); abnormal blood indicators: there were also statistical differences in hypoglycemia, coagulopathy, acidosis, and hyperbilirubinemia (*P* < 0.05). There were 5 cases of premature infants with skin sclerosis in the control group. With admission to the hospital, the hypothermia improved significantly, there was no skin sclerosis in the intervention group, and there was a statistical difference between the two groups (*P* < 0.05).

## 4. Discussion

The thermoregulation efficiency of newborns is much lower than that of adults, and low body temperature or high body temperature are prone to occur. The lower the gestational age and the lower the birth weight, the more immature newborns have a higher risk of hypothermia [12]. Premature babies are immature due to their physiological functions and the development of various organs, and it is easy to cause heat loss and insufficient heat production capacity leading to hypothermia, especially VLBW dumped LBW babies. Premature babies have a large body surface area/weight ratio, and the body has a high proportion of water, less brown fat under the skin, and thin skin, and poor vasomotor constriction can easily cause heat loss. There is little brown fat, and it is difficult to start nontrembling thermogenesis when glucose is not supplemented in time after birth. Both term infants and premature infants lose heat through four methods: evaporation, conduction, convection, and radiation [13, 14]. Due to the abovementioned factors, if you do not take measures to keep your newborn baby warm after birth, body temperature tends to drop rapidly. This study found that, in the full-term infant group, in the control group, 9 cases (9%) had feeding intolerance, and there were 6 cases (6%) in the intervention group, although the incidence of feeding intolerance in the intervention group after effective heat preservation is lower than that in the control group with poor heat preservation; however, no clear statistical difference was found between the control group and the intervention group (*x*^2^ = 0.649,*P* > 0.05). At the same time, we also observed that, in the control group, 5 cases (5%) of gastrointestinal bleeding occurred and 6 cases (6%) in the intervention group, with no statistical difference (*x*^2^ = 0.096,*P* > 0.05). Therefore, this group of experiments believes that, after improving the incidence of hypothermia after birth in full-term infants, the impact on the incidence of full-term infants that are not uncomfortable after birth is not significant [15]. In the premature infant group, 22 cases (22%) had feeding intolerance in the control group, and 8 cases (8%) of feeding intolerance occurred in the intervention group; also, there is a statistical difference between the control group and the intervention group (*x*^2^ = 7.686, *P* < 0.05). At the same time, we also observed that, in the control group, 13 cases (13%) of gastrointestinal bleeding occurred, and *s* cases (5%) occurred in the intervention group, and there are also statistical differences (*x*^2^ = 3.90, *P* < 0.05). Therefore, the research of this subject shows that, the improvement of hypothermia can reduce feeding intolerance after birth of premature infants and occurrence of gastrointestinal bleeding [16].

It is shown that the application of intelligent incubators to newborns not only can improve the various indicators of newborns and reduce the occurrence of adverse reactions but also can improve clinical satisfaction. Analyzing the reasons: in the past, nursing staff used routine care for newborns because the nursing content is relatively simple, and nursing staff monitor the vital signs of newborns, but due to lack of adverse event prevention and emergency response capabilities, newborns are prone to adverse reactions. The intelligent heating box is a new type of nursing model in clinical practice, implementing intelligent incubators for newborns, which can make newborns quickly adapt to the external environment, effectively stabilize its vital signs, improve breathing rate and sleep function, and promote its metabolic function so that newborns can grow up healthy [17, 18]. This is because the intelligent incubator mainly uses bath towels to build a shape similar to a bird's nest for newborns, and putting the newborn in it can make the newborn feel in the mother's body, reduce contact with the outside world, and reduce irritation; therefore, the newborn's body temperature does not change much. Also, by preheating with the help of an incubator, the temperature of the bird's nest can be raised, and it can build a better environment for the development of newborns. In this environment, the newborn's emotions can naturally be effectively soothed; in turn, its heart rate, blood oxygen saturation, and other indicators have been improved; more importantly, the newborn's body temperature does not change much, to a certain extent, its out of the incubator time is shortened, and at the same time, the occurrence of adverse reactions is reduced. Affected by factors such as environment and number of cases, the long-term effects of the two groups need to be supplemented by clinical research analysis [19]. The care of newborns cannot be ignored, seeking a better care plan will promote the faster growth and development of newborns, and maintaining good health is very important [20]. The intelligent heating box imitates the newborn's posture in the mother's body, let the newborn reexperience the feeling of safety and comfort similar to the mother's womb, to some extent, eliminates the tension of the newborn after being separated from the mother, and reduces the strangeness and crying frequency, which is conducive to the growth and development of newborns.

## 5. Conclusions

The application effect of the intelligent heating box in newborn care is exact, can improve parent care satisfaction, increase the sleep time of newborns, reduce body temperature fluctuations, increase blood oxygen saturation, and shorten the time to get out of the incubator, and it is worthy of promotion to reduce the occurrence of complications such as jaundice.

## Figures and Tables

**Table 1:** Comparison of the incidence of hypothermia in term infants and premature infants.

Group	Number of cases (*n*)	Full-term child [*n* (%)]	Premature baby [*n* (%)]
Control group	100	95 (95)	98 (98)
Intervention group	100	37 (37)	49 (49)
*t* (*x*^2^)		(74.95)	(61.63)
*P*		0.000	0.000

**Table 2:** Observation of the incidence of full-term infants.

Project	Control group (*n*)	Intervention group (*n*)	*X* ^2^	*P*
Bradycardia (<100 times/min)	5	3	0.130	0.718
Wet lung	33	16	7.812	0.005
Respiratory failure	11	8	0.523	0.469
Oxygen inhalation	25	12	5.604	0.018
Respiratory pneumonia	8	6	0.307	0.579
RDS	3	1	0.255	0.614
Feeding intolerance	9	6	0.649	0.421
Gastrointestinal bleeding	5	6	0.096	0.756
Hypoglycemia	13	5	3.907	0.048
1 minute or 5 minutes	26	13	5.383	0.020
Apgar <7 minutes				
Metabolic acidosis	16	7	3.979	0.042
Thrombocytopenia	3	2	0.255	0.614
PT or/and APTT extension	13	8	1.330	0.249
HIE	18	12	1.412	0.235
Intracranial hemorrhage	13	8	1.330	0.249
Delayed meconium discharge	23	16	1.561	0.202
Hyperbilirubinemia	42	35	1.035	0.309
HTC increased	9	6	0.649	0.412
Electrolyte disturbance	5	4	0.000	1.000
Pulmonary hemorrhage	1	0	0.000	1.000

**Table 3:** Observation of the incidence of the premature infants group.

Project	Control group (*n*)	Intervention group (*n*)	*X* ^2^	*P*
Bradycardia (<100 beats/min)	13	5	3.907	0.048
Tickle demand	27	11	6.485	0.011
Neonatal wet lung	14	5	4.711	0.030
Respiratory pneumonia	3	4	2.198	0.138
RDS	8	6	0.307	0.579
Respiratory failure	16	11	1.070	0.301
Apnea	13	5	3.907	0.048
Feeding intolerance	22	8	7.686	0.007
Gastrointestinal bleeding	13	5	3.907	0.048
Delayed meconium discharge	27	13	4.528	0.033
Hypoglycemia	23	11	5.103	0.024
1 minute or 5 minutes	23	12	4.190	0.041
Apgar <7 minutes				
Metabolic acidosis	16	6	5.107	0.024
PT or/and APTT extension	22	11	4.391	0.036
Intracranial hemorrhage	18	13	0.954	0.329
Hyperbilirubinemia	63	32	19.268	0.000
Electrolyte disturbance	4	2	0.172	0.687
Thrombocytopenia	3	2	0.000	1.000
HTC increased	7	5	0.355	0.552
Septicemia	4	1	0.821	0.365
NEC	0	0	—	—
Hard and swollen skin	5	0	5.128	0.024
Pulmonary hemorrhage	0	0	—	—

## Data Availability

The data used to support the findings of this study are available from the corresponding author upon request.
